# 1,2,3,5-Tetra­hydro­naphtho­[2,1-*c*]oxepine

**DOI:** 10.1107/S2414314620002886

**Published:** 2020-03-05

**Authors:** Alan J. Lough, Austin Pounder, Christopher Wicks, William Tam

**Affiliations:** a Department of Chemistry, University of Toronto, Toronto, Ontario, M5S 3H6, Canada; b Department of Chemistry, University of Guelph, Guelph, Ontario, N1G 2W1, Canada; University of Aberdeen, Scotland

**Keywords:** crystal structure, ring-opening reaction, regioisomer, weak hydrogen bonds

## Abstract

In the title compound, C_14_H_14_O, the seven-membered ring is in a pseudo-chair conformation. In the crystal, mol­ecules are linked by weak C—H⋯O hydrogen bonds forming layers parallel to (010). In addition, there are weak π–π stacking inter­actions between inversion-related naphthalene ring systems, with a ring centroid–ring centroid distance of 3.518 (5) Å.

## Abstract 2

In the title compound, the seven-membered ring is in a psuedo-chair conformation. In the crystal, mol­ecules are linked by weak C—H⋯O hydrogen bonds forming layers parallel to (010). In addition, there are weak π–π stacking inter­actions between inversion-related naphthalene ring systems.

## Graphical abstract

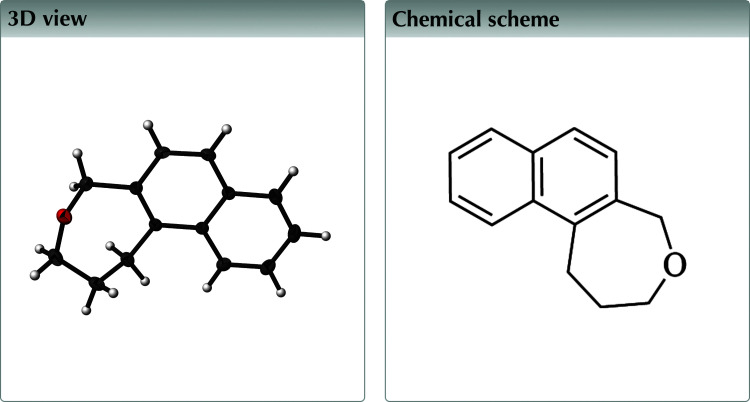

## Structure description

In past years, our research group has investigated the ring-opening reactions of cyclo­propanated oxabenzonorbornadienes (CPOBD) (Carlson *et al.*, 2014 ▸, 2016 ▸, 2018 ▸; Tait *et al.*, 2016 ▸; Tigchelaar *et al.*, 2014 ▸). Recently, we have examined the intra­molecular ring-opening of reaction of CPOBD with tethered alcohol nucleophiles (Wicks *et al.*, 2019 ▸). Based on previous work done in our research group, we anti­cipated two possible modes of ring-opening through nucleophilic attack at either the proximal or distal cyclo­propyl carbon atom. Reaction of the C_1_-alcohol tethered CPOBD **I** (see Fig. 3 ▸) in the presence of *p*-TsOH·H_2_O in toluene afforded the Type 2 **II** and Type 3 **III** ring-opened products in 12% and 59% yields, respectively. The title structure of the Type 2 (**II**) regioisomer was verified by single-crystal X-ray analysis.

**Figure 1:**
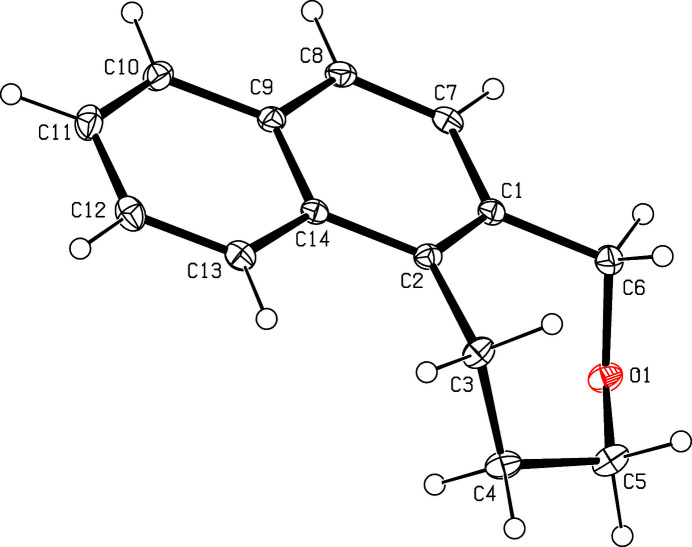
The mol­ecular structure of the title compound with displacement ellipsoids drawn at the 30% probability level.

**Figure 2:**
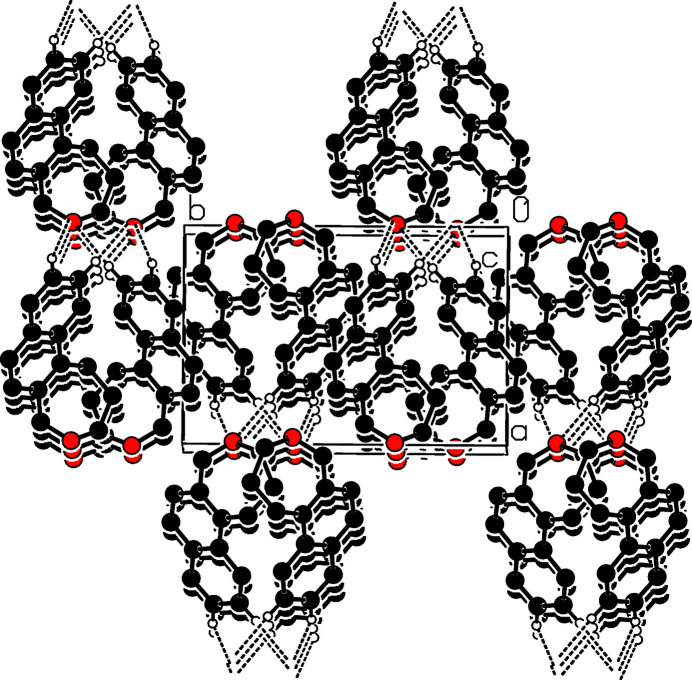
Part of the crystal structure with weak hydrogen bonds shown as dashed lines. Only H atoms involved in hydrogen bonds are shown.

The mol­ecular structure of the title compound is shown in Fig. 1 ▸. The seven-membered ring (C1–C6/O1) is in a pseudo-chair conformation. In the crystal, mol­ecules are linked by weak C—H⋯O hydrogen bonds (Table 1 ▸), forming layers parallel to (010) (Fig. 2 ▸). In addtion, there are weak π–π stacking inter­action between inversion related naphthalene ring systems (C1/C2/C7–C14) with a ring centroid–ring centroid distance of 3.518 (5) Å.

## Synthesis and crystallization

**Figure 3:**
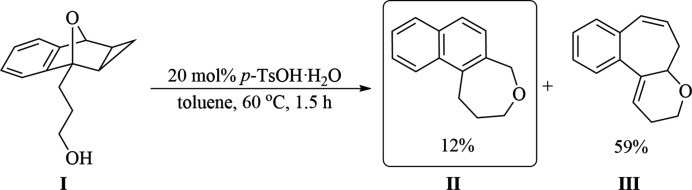
The reaction scheme.

To a 6 dram vial open to air were added the alcohol-tethered cyclo­propanated oxabenzonorbornadiene **I** (0.3547 g, 1.64 mmol), and *p*-TsOH·H_2_O (57.7 mg, 20 mol%) dissolved in 7 ml of toluene (see Fig. 3 ▸). The reaction was left to stir at 333 K for 1.5 h, after which the reaction mixture was cooled and quenched with 10 ml of water. The aqueous layers were combined and back extracted with EtOAc (3 × 5 ml). The organic layers were combined, washed with brine, dried over MgSO_4_, and concentrated *in vacuo*. The resulting crude oil was purified by flash chromatography (EtOAc:hexa­nes, 10: 90) to obtain ring-opened products **II** (38.5 mg, 0.194 mmol, 12%) and **III** (189.8 mg, 0.957 mmol) as a white solid and clear oil, respectively. The title compound **II** was subsequently crystallized from DCM solution by slow evaporation of the solvent to afford colourless blocks.

## Refinement

Crystal data, data collection and structure refinement details are summarized in Table 2 ▸.

## Supplementary Material

Crystal structure: contains datablock(s) I. DOI: 10.1107/S2414314620002886/hb4340sup1.cif

Structure factors: contains datablock(s) I. DOI: 10.1107/S2414314620002886/hb4340Isup2.hkl

Supporting information file. DOI: 10.1107/S2414314620002886/hb4340Isup3.cml

CCDC reference: 1987387

Additional supporting information:  crystallographic information; 3D view; checkCIF report

## Figures and Tables

**Table 1:** Hydrogen-bond geometry (Å, °)

*D*—H⋯*A*	*D*—H	H⋯*A*	*D*⋯*A*	*D*—H⋯*A*
C11—H11*A*⋯O1^i^	0.970 (16)	2.558 (16)	3.4803 (14)	159.0 (11)
C12—H12*A*⋯O1^ii^	0.976 (15)	2.560 (15)	3.4661 (14)	154.5 (11)

Symmetry codes: (i) 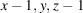 ; (ii) 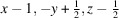 .

**Table 2:** Experimental details

Crystal data	
Chemical formula	C_14_H_14_O
*M*_r_	198.25
Crystal system, space group	Monoclinic, *P*2_1_/*c*
Temperature (K)	150
*a*, *b*, *c* (Å)	9.4559 (3), 12.8405 (4), 8.9638 (3)
β (°)	111.445 (1)
*V* (Å^3^)	1013.02 (6)
*Z*	4
Radiation type	Mo *K*α
μ (mm^−1^)	0.08
Crystal size (mm)	0.37 × 0.26 × 0.25

Data collection	
Diffractometer	Bruker Kappa *APEX* DUO CCD
Absorption correction	Multi-scan (*SADABS*; Krause *et al.*, 2015 ▸)
*T*_min_, *T*_max_	0.723, 0.746
No. of measured, independent and observed [*I* > 2σ(*I*)] reflections	21543, 2347, 2055
*R*_int_	0.021
(sin θ/λ)_max_ (Å^−1^)	0.651

Refinement	
*R*[*F* ^2^ > 2σ(*F* ^2^)], *wR*(*F* ^2^), *S*	0.035, 0.100, 1.07
No. of reflections	2347
No. of parameters	192
H-atom treatment	All H-atom parameters refined
Δρ_max_, Δρ_min_ (e Å^−3^)	0.30, −0.20

Computer programs: *APEX3* and *SAINT* (Bruker, 2018 ▸), *SHELXT2014* (Sheldrick, 2015*a* ▸), *SHELXL2018* (Sheldrick, 2015*b* ▸), *PLATON* (Spek, 2020 ▸) and *publCIF* (Westrip, 2010 ▸).

## full crystallographic data

### Crystal data

**Table:**

C_14_H_14_O	*F*(000) = 424
*M**_r_* = 198.25	*D*_x_ = 1.300 Mg m^−^^3^
Monoclinic, *P*2_1_/*c*	Mo *K*α radiation, λ = 0.71073 Å
*a* = 9.4559 (3) Å	Cell parameters from 9948 reflections
*b* = 12.8405 (4) Å	θ = 2.3–27.5°
*c* = 8.9638 (3) Å	µ = 0.08 mm^−^^1^
β = 111.445 (1)°	*T* = 150 K
*V* = 1013.02 (6) Å^3^	Block, colourless
*Z* = 4	0.37 × 0.26 × 0.25 mm

### Data collection

**Table:**

Bruker Kappa APEX DUO CCD diffractometer	2055 reflections with *I* > 2σ(*I*)
Radiation source: selaed tube with Bruker Triumph monochromator	*R*_int_ = 0.021
φ and ω scans	θ_max_ = 27.6°, θ_min_ = 2.3°
Absorption correction: multi-scan (SADABS; Krause *et al.*, 2015)	*h* = −12→12
*T*_min_ = 0.723, *T*_max_ = 0.746	*k* = −16→16
21543 measured reflections	*l* = −11→11
2347 independent reflections	

### Refinement

**Table:**

Refinement on *F*^2^	Primary atom site location: dual
Least-squares matrix: full	Hydrogen site location: difference Fourier map
*R*[*F*^2^ > 2σ(*F*^2^)] = 0.035	All H-atom parameters refined
*wR*(*F*^2^) = 0.100	*w* = 1/[σ^2^(*F*_o_^2^) + (0.052*P*)^2^ + 0.3073*P*] where *P* = (*F*_o_^2^ + 2*F*_c_^2^)/3
*S* = 1.07	(Δ/σ)_max_ < 0.001
2347 reflections	Δρ_max_ = 0.30 e Å^−^^3^
192 parameters	Δρ_min_ = −0.20 e Å^−^^3^
0 restraints	

### Special details

**Table:**

Geometry. All esds (except the esd in the dihedral angle between two l.s. planes) are estimated using the full covariance matrix. The cell esds are taken into account individually in the estimation of esds in distances, angles and torsion angles; correlations between esds in cell parameters are only used when they are defined by crystal symmetry. An approximate (isotropic) treatment of cell esds is used for estimating esds involving l.s. planes.

### Fractional atomic coordinates and isotropic or equivalent isotropic displacement parameters (Å^2^)

**Table:**

	*x*	*y*	*z*	*U*_iso_*/*U*_eq_	
O1	1.00150 (8)	0.34280 (6)	0.72056 (9)	0.02263 (19)	
C1	0.77678 (11)	0.44489 (7)	0.56216 (11)	0.0171 (2)	
C2	0.65678 (11)	0.37620 (7)	0.53419 (11)	0.0163 (2)	
C3	0.66501 (11)	0.29413 (8)	0.65822 (12)	0.0209 (2)	
H3A	0.5679 (16)	0.2539 (11)	0.6272 (16)	0.029 (3)*	
H3B	0.6758 (16)	0.3299 (11)	0.7608 (17)	0.031 (3)*	
C4	0.79563 (12)	0.21581 (8)	0.68926 (13)	0.0235 (2)	
H4A	0.7759 (16)	0.1550 (11)	0.7461 (16)	0.029 (3)*	
H4B	0.7980 (16)	0.1911 (12)	0.5840 (18)	0.035 (4)*	
C5	0.95069 (12)	0.25805 (9)	0.79119 (14)	0.0254 (2)	
H5A	0.9486 (16)	0.2824 (11)	0.8993 (18)	0.033 (4)*	
H5B	1.0304 (15)	0.2034 (10)	0.8079 (16)	0.025 (3)*	
C6	0.91744 (11)	0.43684 (8)	0.71202 (13)	0.0214 (2)	
H6A	0.9889 (14)	0.4953 (11)	0.7133 (14)	0.023 (3)*	
H6B	0.8913 (15)	0.4415 (10)	0.8106 (17)	0.028 (3)*	
C7	0.77109 (12)	0.52380 (7)	0.45040 (12)	0.0194 (2)	
H7A	0.8559 (16)	0.5724 (11)	0.4711 (16)	0.029 (3)*	
C8	0.64771 (12)	0.53392 (7)	0.31122 (12)	0.0198 (2)	
H8A	0.6454 (15)	0.5891 (11)	0.2355 (16)	0.027 (3)*	
C9	0.52464 (11)	0.46395 (7)	0.27548 (11)	0.0172 (2)	
C10	0.39883 (12)	0.47042 (8)	0.12770 (12)	0.0218 (2)	
H10A	0.4011 (15)	0.5267 (11)	0.0541 (16)	0.026 (3)*	
C11	0.28267 (12)	0.40005 (9)	0.08940 (13)	0.0248 (2)	
H11A	0.1987 (17)	0.4037 (11)	−0.0128 (19)	0.036 (4)*	
C12	0.28495 (12)	0.32073 (9)	0.19878 (13)	0.0236 (2)	
H12A	0.2036 (16)	0.2691 (11)	0.1702 (16)	0.031 (3)*	
C13	0.40269 (11)	0.31364 (8)	0.34385 (12)	0.0201 (2)	
H13A	0.4012 (15)	0.2577 (11)	0.4170 (16)	0.027 (3)*	
C14	0.52804 (10)	0.38377 (7)	0.38733 (11)	0.0157 (2)	

### Atomic displacement parameters (Å^2^)

**Table:**

	*U*^11^	*U*^22^	*U*^33^	*U*^12^	*U*^13^	*U*^23^
O1	0.0183 (4)	0.0229 (4)	0.0269 (4)	0.0028 (3)	0.0085 (3)	0.0040 (3)
C1	0.0174 (4)	0.0164 (4)	0.0174 (5)	0.0016 (3)	0.0064 (4)	−0.0017 (3)
C2	0.0178 (4)	0.0157 (4)	0.0168 (4)	0.0018 (3)	0.0080 (4)	0.0008 (3)
C3	0.0190 (5)	0.0241 (5)	0.0207 (5)	0.0002 (4)	0.0086 (4)	0.0058 (4)
C4	0.0254 (5)	0.0193 (5)	0.0274 (5)	0.0016 (4)	0.0115 (4)	0.0069 (4)
C5	0.0217 (5)	0.0276 (6)	0.0268 (5)	0.0052 (4)	0.0088 (4)	0.0092 (4)
C6	0.0197 (5)	0.0203 (5)	0.0215 (5)	0.0003 (4)	0.0043 (4)	−0.0025 (4)
C7	0.0215 (5)	0.0142 (4)	0.0239 (5)	−0.0025 (4)	0.0099 (4)	−0.0024 (4)
C8	0.0260 (5)	0.0143 (4)	0.0207 (5)	0.0010 (4)	0.0105 (4)	0.0016 (4)
C9	0.0190 (5)	0.0160 (4)	0.0175 (5)	0.0034 (4)	0.0077 (4)	−0.0004 (3)
C10	0.0231 (5)	0.0234 (5)	0.0185 (5)	0.0061 (4)	0.0070 (4)	0.0015 (4)
C11	0.0192 (5)	0.0324 (6)	0.0197 (5)	0.0048 (4)	0.0035 (4)	−0.0043 (4)
C12	0.0177 (5)	0.0265 (5)	0.0265 (5)	−0.0023 (4)	0.0080 (4)	−0.0074 (4)
C13	0.0194 (5)	0.0195 (5)	0.0231 (5)	−0.0011 (4)	0.0099 (4)	−0.0025 (4)
C14	0.0167 (4)	0.0151 (4)	0.0170 (4)	0.0017 (3)	0.0081 (4)	−0.0016 (3)

### Geometric parameters (Å, º)

**Table:**

O1—C5	1.4277 (13)	C6—H6B	1.003 (14)
O1—C6	1.4323 (12)	C7—C8	1.3675 (14)
C1—C2	1.3858 (13)	C7—H7A	0.979 (14)
C1—C7	1.4120 (14)	C8—C9	1.4112 (14)
C1—C6	1.5092 (13)	C8—H8A	0.975 (14)
C2—C14	1.4327 (13)	C9—C10	1.4229 (13)
C2—C3	1.5131 (13)	C9—C14	1.4292 (13)
C3—C4	1.5372 (14)	C10—C11	1.3657 (16)
C3—H3A	1.000 (14)	C10—H10A	0.984 (14)
C3—H3B	0.999 (14)	C11—C12	1.4084 (16)
C4—C5	1.5163 (15)	C11—H11A	0.969 (15)
C4—H4A	0.987 (14)	C12—C13	1.3712 (15)
C4—H4B	1.004 (15)	C12—H12A	0.976 (14)
C5—H5A	1.025 (15)	C13—C14	1.4248 (13)
C5—H5B	1.000 (13)	C13—H13A	0.977 (14)
C6—H6A	1.007 (13)		
			
C5—O1—C6	113.34 (8)	O1—C6—H6B	108.3 (8)
C2—C1—C7	120.80 (9)	C1—C6—H6B	111.0 (8)
C2—C1—C6	120.95 (9)	H6A—C6—H6B	109.0 (10)
C7—C1—C6	118.24 (9)	C8—C7—C1	120.93 (9)
C1—C2—C14	119.18 (9)	C8—C7—H7A	118.6 (8)
C1—C2—C3	119.45 (9)	C1—C7—H7A	120.5 (8)
C14—C2—C3	121.38 (8)	C7—C8—C9	120.28 (9)
C2—C3—C4	114.25 (8)	C7—C8—H8A	119.9 (8)
C2—C3—H3A	111.1 (8)	C9—C8—H8A	119.8 (8)
C4—C3—H3A	107.9 (8)	C8—C9—C10	120.90 (9)
C2—C3—H3B	108.5 (8)	C8—C9—C14	119.61 (9)
C4—C3—H3B	109.2 (8)	C10—C9—C14	119.47 (9)
H3A—C3—H3B	105.5 (11)	C11—C10—C9	121.14 (10)
C5—C4—C3	114.27 (9)	C11—C10—H10A	122.0 (8)
C5—C4—H4A	107.2 (8)	C9—C10—H10A	116.8 (8)
C3—C4—H4A	108.7 (8)	C10—C11—C12	119.82 (10)
C5—C4—H4B	109.3 (8)	C10—C11—H11A	120.8 (9)
C3—C4—H4B	109.2 (8)	C12—C11—H11A	119.4 (9)
H4A—C4—H4B	108.0 (12)	C13—C12—C11	120.57 (10)
O1—C5—C4	114.42 (9)	C13—C12—H12A	119.5 (8)
O1—C5—H5A	108.2 (8)	C11—C12—H12A	119.9 (8)
C4—C5—H5A	109.3 (8)	C12—C13—C14	121.55 (10)
O1—C5—H5B	104.1 (8)	C12—C13—H13A	118.7 (8)
C4—C5—H5B	110.4 (8)	C14—C13—H13A	119.7 (8)
H5A—C5—H5B	110.3 (11)	C13—C14—C9	117.41 (9)
O1—C6—C1	113.36 (8)	C13—C14—C2	123.42 (9)
O1—C6—H6A	105.7 (7)	C9—C14—C2	119.15 (8)
C1—C6—H6A	109.2 (7)		
			
C7—C1—C2—C14	2.09 (14)	C7—C8—C9—C14	1.43 (14)
C6—C1—C2—C14	−177.41 (8)	C8—C9—C10—C11	177.03 (9)
C7—C1—C2—C3	−178.58 (9)	C14—C9—C10—C11	−1.35 (15)
C6—C1—C2—C3	1.92 (14)	C9—C10—C11—C12	1.34 (15)
C1—C2—C3—C4	−62.32 (12)	C10—C11—C12—C13	0.19 (16)
C14—C2—C3—C4	117.00 (10)	C11—C12—C13—C14	−1.72 (15)
C2—C3—C4—C5	76.33 (12)	C12—C13—C14—C9	1.66 (14)
C6—O1—C5—C4	69.71 (12)	C12—C13—C14—C2	−176.93 (9)
C3—C4—C5—O1	−63.49 (12)	C8—C9—C14—C13	−178.54 (9)
C5—O1—C6—C1	−87.27 (10)	C10—C9—C14—C13	−0.14 (13)
C2—C1—C6—O1	65.29 (12)	C8—C9—C14—C2	0.11 (13)
C7—C1—C6—O1	−114.22 (10)	C10—C9—C14—C2	178.51 (8)
C2—C1—C7—C8	−0.56 (15)	C1—C2—C14—C13	176.72 (9)
C6—C1—C7—C8	178.95 (9)	C3—C2—C14—C13	−2.60 (14)
C1—C7—C8—C9	−1.23 (15)	C1—C2—C14—C9	−1.84 (13)
C7—C8—C9—C10	−176.95 (9)	C3—C2—C14—C9	178.83 (8)

### Hydrogen-bond geometry (Å, º)

**Table:**

*D*—H···*A*	*D*—H	H···*A*	*D*···*A*	*D*—H···*A*
C11—H11*A*···O1^i^	0.970 (16)	2.558 (16)	3.4803 (14)	159.0 (11)
C12—H12*A*···O1^ii^	0.976 (15)	2.560 (15)	3.4661 (14)	154.5 (11)

Symmetry codes: (i) *x*−1, *y*, *z*−1; (ii) *x*−1, −*y*+1/2, *z*−1/2.
